# Robustness of a Topologically Protected Surface State in a Sb_2_Te_2_Se Single Crystal

**DOI:** 10.1038/srep36538

**Published:** 2016-11-18

**Authors:** Chao-Kuei Lee, Cheng-Maw Cheng, Shih-Chang Weng, Wei-Chuan Chen, Ku-Ding Tsuei, Shih-Hsun Yu, Mitch Ming-Chi Chou, Ching-Wen Chang, Li-Wei Tu, Hung-Duen Yang, Chih-Wei Luo, Marin M. Gospodinov

**Affiliations:** 1 Department of Photonics, National Sun Yat-sen University, 70, Lienhei Road, Kaohsiung 80424, Taiwan; 2 Department of Physics, National Sun Yat-sen University, 70, Lienhei Road, Kaohsiung 80424, Taiwan; 3 National Synchrotron Radiation Research Center, Hsinchu 30076, Taiwan; 4 Department of Materials and Optoelectronics Science, National Sun Yat-sen University, 70, Lienhei Road, Kaohsiung 80424, Taiwan; 5 Department of Medical Laboratory Science and Biotechnology, Kaohsiung Medical University, Kaohsiung 80708, Taiwan; 6 Department of Electrophysics, National Chiao Tung University, Hsinchu 300, Taiwan; 7 Institute of Solid State Physics, Bulgarian Academy of Sciences, Blvd. TzarigradskoChaussee 72, 1784 Sofia, Bulgaria

## Abstract

A topological insulator (TI) is a quantum material in a new class with attractive properties for physical and technological applications. Here we derive the electronic structure of highly crystalline Sb_2_Te_2_Se single crystals studied with angle-resolved photoemission spectra. The result of band mapping reveals that the Sb_2_Te_2_Se compound behaves as a *p*-type semiconductor and has an isolated Dirac cone of a topological surface state, which is highly favored for spintronic and thermoelectric devices because of the dissipation-less surface state and the decreased scattering from bulk bands. More importantly, the topological surface state and doping level in Sb_2_Te_2_Se are difficult to alter for a cleaved surface exposed to air; the robustness of the topological surface state defined in our data indicates that this Sb_2_Te_2_Se compound has a great potential for future atmospheric applications.

A topological insulator (TI) is a quantum material in a new class with attractive properties for physical and technological applications because of its potential in spintronic devices^1^,^2^,^3^,^4^,^5^,^6^. Two-dimensional (2D) TI are typically associated with gapless edge states; three-dimensional TI are associated with gapless surface states. The edge and surface states in these systems generally reveal a Dirac-cone-like dispersion; the spin degeneracy of Dirac fermions in these states of TI is removed and locked to the momentum, as these surface states of 3D TI exhibit a helical spin structure with the spin vectors parallel to the surface and perpendicular to momentum *k*. This unique topological phase offers a platform to realize diverse quantum phenomena, such as a topological magnetoelectric effect^7^, a Majorana fermion^8^, the quantum spin-Hall effect^9^ and spintronic applications^10^.

A key to develop applications in topological quantum computing^7^,^11^ and low- power spintronic devices^10^ is that the dissipation-less surface states must be in the topological transport regime; specifically, the presence of an isolated Dirac cone is highly favoured because of the decreased scattering from bulk bands. Manipulating the position of the Dirac point in TI for future applications becomes a crucial problem. TI in the bismuth family – Bi_2_Se_3_, Bi_2_Te_3_ and Sb_2_Te_3_ – have a tetradymite structure, which consists of covalently bonded quintuple layers (QL) with a weak interaction through a van der Waals force within QL^12^. Bi_2_Se_3_ and Bi_2_Te_3_ have the chemistry of a non-stoichiometric compound^13^,^14^; both compounds are invariably doped through the crystal defects, which results in the transport properties being affected by bulk carriers. A single crystal of Bi_2_Se_3_ invariably exhibits a *n*-type doping behaviour because of the Se vacancies or anti-site defects^15^,^16^. Regarding the Bi_2_Te_3_ compound, both *n*-type and *p*-type behaviour can be achieved on varying the initial composition to become either Bi-rich or Te-rich depending on growth conditions, which results in Te and Bi anti-site defects, but a Dirac point in Bi_2_Te_3_ immersed in the bulk valence band can cause strong scattering from bulk carriers. In contrast, the Sb_2_Te_3_ compound invariably behaves as a *p*-type semiconductor^17^,^18^,^19^. The position of the Dirac point is inherently located above the Fermi level, but the bulk valence band observed across the Fermi level in direction 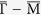 is unfavourable for an application of spintronic devices^18^,^19^,^20^, even though recent work demonstrated that the position of the chemical potential can be varied with the thickness of Sb_2_Te_3_ thin films^21^. Additionally, Sb_2_Te_3_ is an important *p*-type element in thermoelectric modules. In early work, Hicks and Dresselhaus proposed that low-dimensional systems could enhance the thermoelectric efficiency through an enlarged thermopower^22^, but a decreased thermopower value was observed in Sb_2_Te_3_ thin films; the discrepancy was resolved in recent work on Sb_2_Te_3_ thin films^23^,^24^,^25^. The separate contributions to thermopower from the bulk and the surface could cause a decreased total thermopower^26^. A surface state existing in the energy gap with a small concentration of bulk carriers could benefit an enhanced thermopower.

Authors of several theoretical and experimental works have focused on the predictions and findings of an isolated Dirac cone to decrease scattering from bulk carriers in ternary tetradymite-like topological insulators, such as Sb_*x*_Bi_2-*x*_Se_2_Te and Bi_2-*x*_Sb_*x*_Te_3-*y*_Se_*y*_ systems^27^,^28^,^29^,^30^,^31^,^32^,^33^, but experimental work on *p*-type Sb_2_Te_3-*x*_Se_*x*_ ternary topological insulators is lacking. A calculation indicated that a topological surface state still existed for *x* ≈ 0.94^27^. In previous work, only *p*-type doped behaviour in Sb_2_Te_2_Se was proposed^34^. A recent observation of the Zeeman effect in the topological surface state of Sb_2_Te_2_Se was reported^35^; the *g*-factor of the topological surface state extracted from the Zeeman shift is disparate in Bi_2_Se_3_ and Sb_2_Te_2_Se, but no experimental result for the band structure of Sb_2_Te_2_Se has been reported.

In the present work, we measured angle-resolved photoemission spectra (ARPES) of highly crystalline Sb_2_Te_2_Se and Sb_2_Te_3_ single crystals. According to the measured band structure of Sb_2_Te_2_Se, an examination of angle-resolved photoemission spectra in an experiment dependent on photon energy indicated that the 2D behaviour of the topological surface state was unaffected. A maximum of the bulk valence band (BVB) near the Fermi level appeared in the middle of direction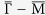, but no clear crossing at the Fermi level was observed. In direction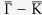, no contribution about the Fermi level from the BVB was found; only a few contributions about the Γ point from the BVB in the Sb_2_Te_2_Se compound appeared. More importantly, we found that the doping level of Sb_2_Te_2_Se, unlike compounds Bi_2_Se_3_ and Bi_2_Te_3_^34^,^36^, is difficult to vary, even for a cleaved surface exposed to air. The robustness of a topological surface state in Sb_2_Te_2_Se benefits the control of electrical properties of fabricated devices. These results indicate that Sb_2_Te_2_Se can be an effective candidate to improve the performance of future spintronic and thermoelectric devices.

## Results

### Electronic structure of Sb_2_Te_2_Se

**Figure 1:**
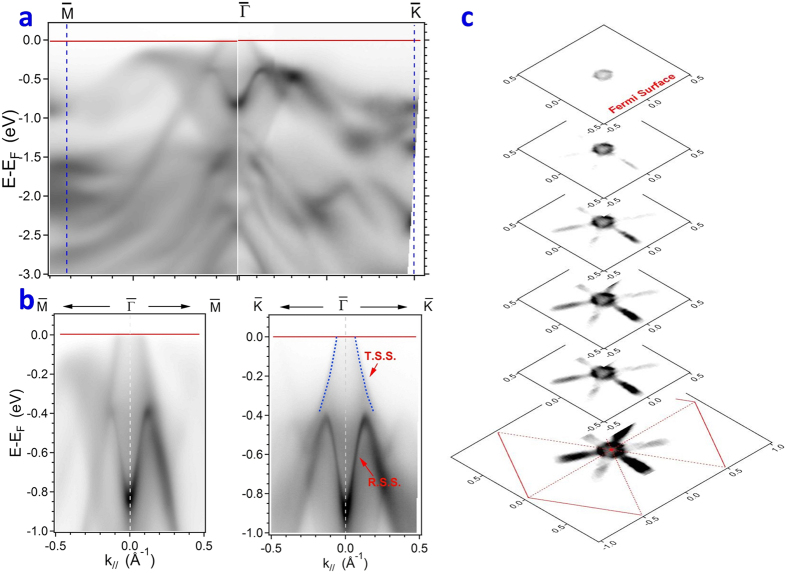
Band mapping of Sb_2_Te_2_Se in a wide energy range using photon energy 24 eV. (**a**) Band structure of Sb_2_Te_2_Se(0001) along directions 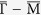 and 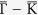. (**b**) Enlarged band structure about the *Γ* point of (**a**,**c**) Plots of photoemission intensity in momentum space at various constant energies at photon energy 24 eV. The order of slices from the top slice is the Fermi level, 30, 60, 90, 120 and 150 meV. Only a circular-like surface state is observed at the Fermi surface.

In a Sb-based ternary tetradymite-like topological insulator, Sb_2_Te_2_Se naturally crystallizes in a chalcogen-ordered structure with a Te-Sb-Se-Sb-Te QL, and has the stoichiometric composition of these materials. In the stacking order, the inversion symmetry is unbroken; the topological surface state persists. Highly crystalline single crystals of Sb_2_Te_2_Se and Sb_2_Te_3_ were grown in a home-made resistively heated floating-zone (RHFZ) furnace. X-ray diffraction (XRD) and Raman scattering were applied to characterize the crystal quality (see Supplementary materials). The electronic structure of a Sb_2_Te_2_Se single crystal was derived using high-resolution ARPES. Figure 1a displays the band structure of Sb_2_Te_2_Se(0001) on a large energy scale along directions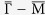 and 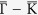; the spectra were recorded at photon energy 24 eV. In direction 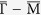, the BVB disperses toward the Fermi level in the middle of branch 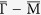. The band maximum of BVB about *k*_//_ ~ 0.4 Å^−1^ was located near the Fermi level, but no band crossing at the Fermi level was observed in the analysis of the momentum- distribution curves (MDC). In branch 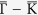, no component of the bulk band projection appeared in the range of the Fermi level and 0.35 eV. A ‘M’-shaped resonant surface state (RSS) about the *Γ* point similar to that in Sb_2_Te_3_ was observed in the bulk band gap within the scale of binding energy 0.40 to 0.86 eV. The topological surface state (TSS) appears at binding energy 0.40 eV and disperses toward the Fermi level with *k*_*F*_ = 0.065 Å^−1^ extracted from the MDC. A sharp topological surface state with width of MDC about 0.047 Å^−1^ indicates that the band-mapping result shows a highly crystalline quality of the present samples. The entire measured band structure agrees qualitatively with previously calculated results^28^,^37^ and behaves in a typical *p*-type manner. In comparison with the band structure of Sb_2_Te_3_^20^, the contribution of BVB of Sb_2_Te_2_Se around the Fermi level in the middle of branch 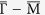 in our result and calculation show a strongly suppressed behaviour. The calculated result shows also a slight contribution of BVB from the bulk band projection around the Fermi level at the Γ point; an ARPES experiment dependent on photon energy was conducted to examine the contribution of BVB around the Γ point. We noticed that the energy scale between the Fermi level and the TSS in our result differs slightly from that in a preceding calculation.

Figure 1b displays the enlarged band structure about the *Γ* point along directions 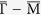 and 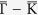. Along direction 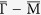, the maximum of the BVB in the middle of direction 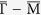 approaches the Fermi level and has a band edge about binding energy 40 meV, which is determined from a series of energy distribution curves (EDC) about *k*_//_ = 0.4 Å^−1^ (see Supplementary materials). Along direction 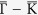, the contribution from the BVB is observed only below binding energy 0.35 eV. The topological surface state with a linear dispersion down to binding energy 0.22 eV can be extracted from the result of fitting the MDC. To confirm the existence of the Dirac point, we performed a doping test with alkali-metal-doped Sb_2_Te_2_Se (see Supplementary materials). The TSS and RSS were clearly moved to a large binding energy after deposition of potassium or caesium atoms in small proportions, which implied a *n*-doping effect from the alkali-metal atoms. Once the shift magnitude approached 83 meV, the doping effect became saturated; the TSS and RSS no longer moved toward the region of large binding energy. The band dispersion of TSS still shows a linear dispersion after doping with an alkali metal, but no crossing point is observed because of the saturation of the doping effect. The position of the Dirac point was determined at 237 meV above the Fermi level through an extrapolation of the linear dispersion of TSS. We recorded infrared (FTIR) spectra to detect the band gap in Sb_2_Te_2_Se. A band gap ~ 417 meV at 98 K was extracted from the absorption edge in the infrared spectrum (see Supplementary materials). This result indicates that an isolated Dirac cone with the Dirac point located at 237 meV above the Fermi level exists in the Sb_2_Te_2_Se compound. Our observation shows also a consistency with the recent result of the Landau-level (LL) spectra in Sb_2_Te_2_Se^35^. Additionally, the scattering from bulk carriers is strongly suppressed. Figure 1c shows plots of the photoemission intensity in momentum space with various constant energies at the Fermi level −30, 60, 90, 120 and 150 meV; the spectra are recorded at photon energy 24 eV. Only a circular-like surface state is observed on the Fermi surface. At 30 and 60 meV, the bulk component appears in direction 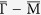 and shows a three-fold symmetry. With increasing binding energy, a slight deformation of the TSS is observed in the images of constant energy mapping.

**Figure 2:**
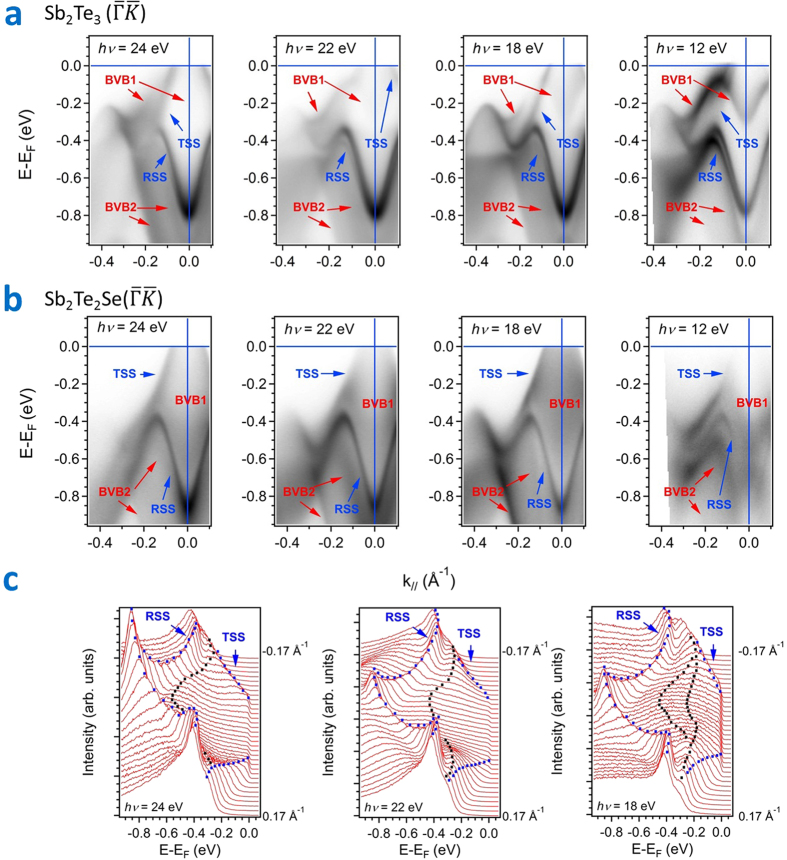
Comparison of the electronic structure of Sb_2_Te_3_ and Sb_2_Te_2_Se along dection 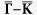 on a photon-energy-dependent experiment. (**a**) Band-mapping results of ARPES in Sb_2_Te_3_ (0001) along direction 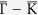 with photon energies 24, 22, 18 and 12 eV. (**b**) Band-mapping results of ARPES in Sb_2_Te_2_Se (0001) along direction 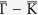 with photon energies 24, 22, 18 and 12 eV. (**c**) EDC about the Γ point in a series with photon energies 24, 22 and 18 eV.

In the photoemission experiment, the normal component of electron wave vector *k*_⊥_ of the initial state is based on a free-electron model in the final state^38^,


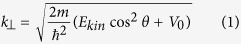


in which *E*_*kin*_ is the kinetic energy of the photoelectron and *V*_0_ is the inner potential. To examine the property of a 3D bulk valence band, we conducted an ARPES experiment dependent on photon energy in a range from 12 to 42 eV, to cross several periods of the Brillouin zone (BZ) in the *k*_*z*_ direction if the inner potential is assumed to be *V*_0_ = 9.64 eV^39^. A *k*-space map in the bulk BZ plotted with varied photon energy at collecting angle ±18° of the detector was used to explore the *k*_*z*_ dependence of TSS, RSS and bulk bands (see Supplementary materials). The electronic structure of Sb_2_Te_3_ was studied for comparison with the same Sb_2_Te_2_Se crystal. Figure 2a shows the band-mapping results of ARPES spectra in Sb_2_Te_3_ (0001) along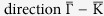 with photon energies 24, 22, 18 and 12 eV. The Rashba splitting in RSS is observed clearly in the plots at 22, 18 and 12 eV. The extracted *k*_*F*_ ~ 0.065 Å^−1^ and *v*_F_ of TSS ~2.32 eV Å are consistent with previous work on Sb_2_Te_3_^40^. The connection between TSS and BVB1 proposed in preceding work is seen also in the plot at 22 and 12 eV. Figure 2b shows band-mapping results of ARPES spectra in Sb_2_Te_2_Se (0001) in a series along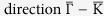 with photon energies 24, 22, 18 and 12 eV. In contrast to the results of Sb_2_Te_3_ along direction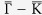, BVB1 and TSS show only a weak interaction about the *Γ* point from BVB1 in the range about 0.35 eV binding energy, as shown in plots at 22 and 18 eV. No altered band dispersion in TSS and RSS was observed in the experiment with varied photon energy, indicating a 2-D behavior. Other results dependent on photon energy show the same results as in Fig. 2 (see Supplementary materials). In the present data, the width of MDC in RSS is about 0.072 Å^−1^; this value is small, and no clear splitting of RSS is observed. These conditions imply only a weak spin-orbital interaction in the Sb_2_Te_2_Se compound; the momentum resolution in the present data is insufficient to resolve the predicted Rashba splitting. The reason is a weak spin-orbital interaction in Sb_2_Te_2_Se compound because of light element Se^41^. Figure 2c displays EDC about the Γ point in a series with photon energies 24, 22 and 18 eV. An analysis of MDC and EDC near the Fermi level around the Γ point with 18, 22, 30 and 32 eV was conducted to examine the band crossing at the Fermi level. Except the TSS, no obvious bulk band disperses and crosses the Fermi level (see Supplementary materials). At photon energy 18 eV, a clear intensity between the surface state at the Γ point was observed, but the same intensity at the next BZ was not observed in the plots at 30 and 32 eV when we verified the period of the *k*_*z*_ dependence (see Supplementary materials). For comparison with the result of the LL spectra^35^, no feature therein was observed below sample bias 150 mV, indicating that the observation of the shaded area between the surface state in the plot of 18 eV might come from the bulk band projection, even though we found no clear band dispersion around at the Γ point.

**Figure 3:**
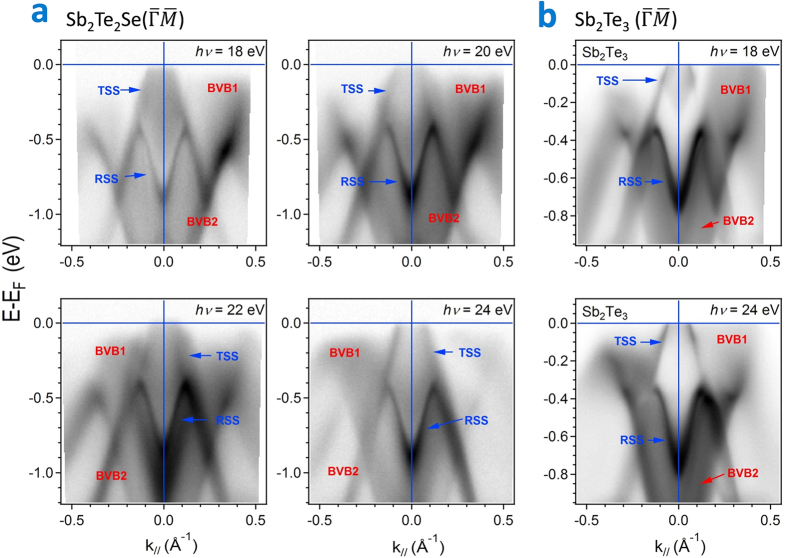
Comparison of the electronic structures of Sb_2_Te_2_Se and Sb_2_Te_3_ along direction 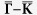 on a photon-energy-dependent experiment. (**a**) Band-mapping results of ARPES spectra in Sb_2_Te_2_Se (0001) along direction 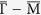 with photon energies 18, 20 22 and 24 eV. (**b**) Band-mapping results of ARPES in Sb_2_Te_3_(0001) along direction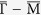. with photon energies 18 and 24 eV.

**Figure 4:**
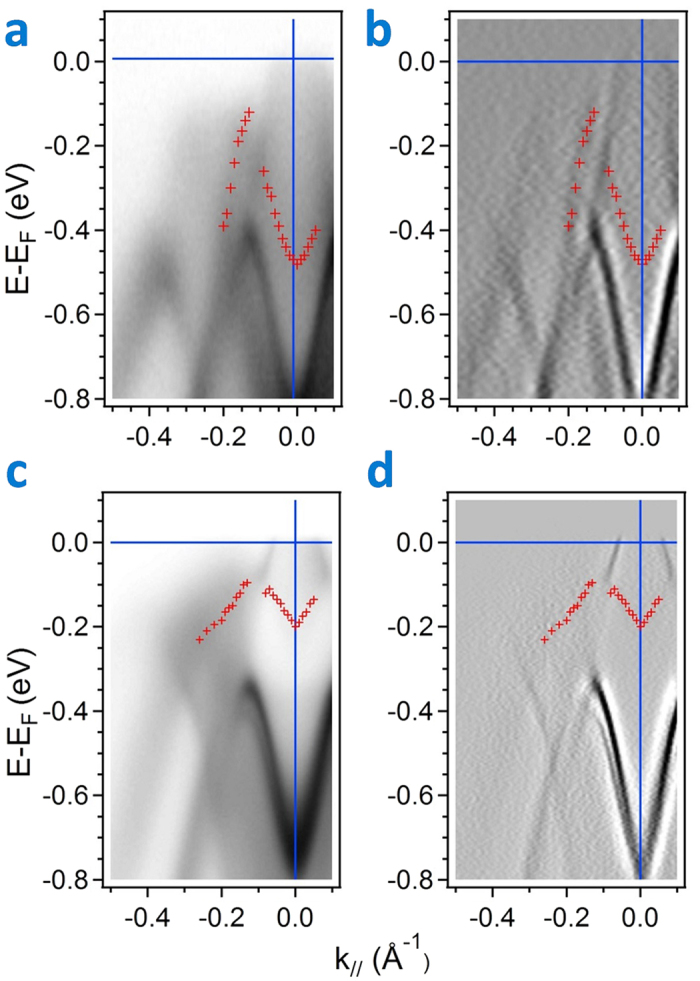
High-resolution ARPES near the Fermi level in Sb_2_Te_2_Se and Sb_2_Te_3_ at photon energy 22 eV. (**a**) Photoemission intensity image of ARPES in Sb_2_Te_2_Se(0001) recorded at photon energy 22 eV. (**b**) Second derivative plot of (**a,c**), Photoemission intensity image of ARPES in Sb_2_Te_3_(0001) recorded at photon energy 22 eV. (**d**) Second derivative plot of (**c**).

Figure 3a displays plots of the band-mapping results in Sb_2_Te_2_Se (0001) along 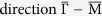 with photon energies 18, 20, 22 and 24 eV. The bulk valence band disperses toward the Fermi level and has a band maximum about the middle of 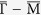. According to the analysis of MDC at the Fermi level and EDC with varied photon energy, no clear band crossing at the Fermi level is observed in a range covering 42 eV (see Supplementary materials). Figure 3b shows plots of the band-mapping results in Sb_2_Te_3_ along 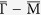; these results are similar to those reported^18^,^19^,^40^. The BVB1 in Sb_2_Te_3_ has a clear band crossing at the Fermi level in the middle of  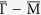, which indicates that scattering from bulk carriers is greater than that in the Sb_2_Te_2_Se compound. Regarding the interaction of BVB and TSS about the *Γ* point, Fig. 4a,b display the photoemission intensity image of ARPES and the second derivative of the image in Sb_2_Te_2_Se respectively; these spectra were recorded at photon energy 22 eV. The BVB1 band appears to have a crossing with the TSS band. This result implies an orbital hybridization that can occur about the *Γ* point to be similar to that in previous work on Sb_2_Te_3_^40^. A comparison of the photoemission intensity image in Sb_2_Te_3_ along direction 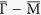 is shown in Fig. 4c,d. The Rashba splitting of TSS in Sb_2_Te_3_ is clearly observed in the plot of the second derivative of the photoemission intensity image, but in our spectra no splitting in Sb_2_Te_2_Se can be resolved. Additionally, in contrast to Sb_2_Te_3_, because a major contribution of charge carriers in Sb_2_Te_2_Se comes only from the BVB about the Γ point and because no other contribution is observed in BZ, the resistivity in Sb_2_Te_2_Se is expected to be larger than that in Sb_2_Te_3_.

The dependence of resistivity on temperature and Hall measurement at room temperature were measured, showing the resistivity in Sb_2_Te_2_Se to be ten times that in Sb_2_Te_3_ (see Supplementary materials). The charge carrier number extracted from a Hall measurement near 296 K is 2.2 × 10^20^ cm^−3^ for Sb_2_Te_3_ and 3.1 × 10^19^ cm^−3^ for Sb_2_Te_2_Se. A charge carrier number ~ 3.1 × 10^19^ cm^−3^ in Sb_2_Te_2_Se indicates that it behaves as a heavily doped semiconductor, but the bulk carrier number in Sb_2_Te_2_Se still decreases significantly. These results show the consistency of our ARPES result. Because the position of the Dirac point can be manipulated on controlling the chemical composition of a topological insulator in the bismuth family TIs, a possibility to decrease the charge carrier number is to increase the content of Se atoms.

The efficiency of a thermoelectric cooler is generally described according to the value of the figure of merit, *Z*_*T*_, expressed as^42^,


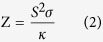


in which *S* is thermopower, *σ* is electrical conductivity and *κ* is thermal conductivity. To consider a two-band model of a TI, the total thermopower and electrical conductivity come from the surface state and the bulk carriers. The total electrical conductivity is expressible as *σ*_*total*_ = *σ*_*bulk*_ + *σ*_*surface*_; the total thermopower is expressed as *S*_*total*_ = (*σ*_*bulk*_*S*_*bulk*_ + *σ*_*surface*_*S*_*surface*_)/*σ*_*total*_^26^. In a thin-film system, the contribution of electrical conductivity from bulk bands can be decreased on controlling the thickness of the TI. As Sb_2_Te_3_ behaves as a *p*-type semiconductor, the maximum of the valence band is above the position of the Dirac point. The sign of the thermopower from the surface state and bulk are opposite if the position of the chemical potential is located above the Dirac point, which can result in a decreased thermopower. In contrast, more bulk carriers can increase the part of the thermal conductivity from the charge carriers if the position of the chemical potential is located below the Dirac point. Although the thermopower is increased, a greater thermal conductivity from bulk charged carriers confers no benefit to enhance the figure of merit. In our present Sb_2_Te_2_Se system, the maximum of the valence band is suppressed inherently below the Fermi level, which implies the same sign of the thermopower for charge carriers from the surface state and from the bulk valence band. Sb_2_Te_2_Se has a high potential as a *p*-type thermoelectric element, especially in an application of a hybrid thin-film heterostructure.

### Robustness of a topological surface state

**Figure 5:**
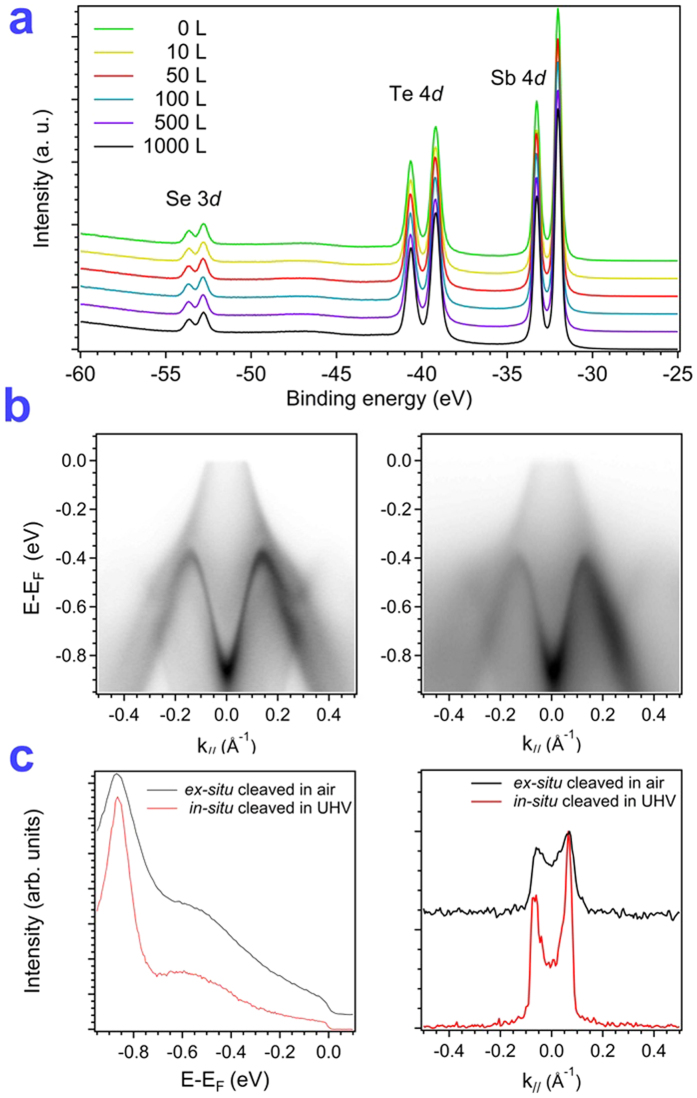
Robustness of TSS in oxygen and atmospheric environments. (**a**) Core-level spectra of Sb_2_Te_2_Se after deposition of oxygen. (**b**) Comparison of photoemission plots recorded at 24 eV for samples cleaved *in situ* in UHV and cleaved *ex situ* in air. (**c**) Comparison of EDC in the normal emission and MDC at the Fermi level. The feature of RSS becomes broadened but the peak position is constant. The same values of *k*_*F*_ extracted from MDC for both cleaving conditions implies that no impurity doping occurs for a sample cleaved in an atmospheric environment.

Surface reactivity is a crucial problem for a TI in the bismuth family, such as Bi_2_Se_3_ and Bi_2_Te_3_. Previous work on the degradation of the surfaces of Bi_2_Se_3_ and Bi_2_Te_3_ shows that the doping level can be altered with gas or vapor contamination^34^,^36^,^43^,^44^,^45^; an extrinsic doping dependent on ambient environmental conditions in Sb_2_Te_3_ was also studied with Hall measurements^46^; this effect limits the potential applications in TI in the bismuth family. In the present work, experiments involving the deposition of oxygen and an air environment were performed to detect the robustness of the topological surface state in Sb_2_Te_2_Se. Figure 5a displays the resolution of core-level photoemission for a surface cleaved *in situ* and exposed to varied content of oxygen. No oxidation signal was observed after the deposition of oxygen about 1000 Langmuir (L) near 296 K. The band dispersions and the Fermi level-crossing vector of TSS and RSS before and after exposure were the same, which implies no impurity doping on the surface. Figure 5b displays the results of photoemission-mapping images after cleaving *in situ* in UHV and *ex situ* in an air environment. According to a comparison of these results, the image for the sample cleaved *ex situ* is only slightly smeared because of the surface pollution, but no change of the band position occurs, as shown in Fig. 5c. This result reveals the robustness of TSS in a Sb_2_Te_2_Se compound and indicates that this Sb_2_Te_2_Se compound has a high potential for applications in spintronic devices. In particular, the authors of a recent investigation proposed the possibility of tuning the doping level on varying the thickness of Sb_2_Te_3_^21^. Further work on Sb_2_Te_2_Se becomes important.

## Conclusion

For Sb_2_Te_2_Se single crystals of high quality that we grew, we studied the electronic structure of the Sb_2_Te_2_Se compound with ARPES. The results of band mapping reveal that this Sb_2_Te_2_Se compound is a *p*-type semiconductor and has an isolated Dirac cone. That the maximum of the bulk valence band in the middle of  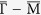 is suppressed below the Fermi level implies a possibility of decreased scattering from that bulk valence band and that the thermopower can have the same sign for the surface state and the bulk carriers; the Sb_2_Te_2_Se system is hence a potential candidate for the future application of spintronic and thermoelectric devices. More importantly, the robustness of TSS in the Sb_2_Te_2_Se system benefits the control of the performance of the devices.

## Methods

### Crystal growth

Single crystals of Sb_2_Te_2_Se and Sb_2_Te_3_ were grown in a home-made resistively heated floating-zone furnace (RHFZ). The initial raw materials Sb_2_Te_2_Se and Sb_2_Te_3_ were mixed according to the stoichiometric ratios. The stoichiometric mixtures of highly pure elements Sb (99.995%), Se (99.995%) and Te (99.995%) were first melted at 850~950 °C and cooled to about 23 °C afterward in an evacuated silica tube. The material was used as a feeding rod for the following RHFZ experiment. The growth rate was 1.5 mm/h. After growth, the crystals were cooled to 23 °C over 50 h. Crystals of diameter 3.0 mm and length 30 mm were obtained reproducibly. A crystal cleaved along the basal plane showed a silvery, shiny, mirror-like surface.

### ARPES

ARPES were measured at National Synchrotron Radiation Research Center in Hsinchu, Taiwan at beamline BL21B1 U9-CGM for spectroscopy. The photoemission spectra were measured in a UHV chamber equipped with a hemispherical analyzer (Scienta R4000, collecting angle ±15°). The polarization vector was invariably in the angular dispersive plane. The single crystals were cleaved *in situ* and measured at a base pressure 5.1 × 10^−11^ Torr. All spectra were recorded for samples at 83 K and with varied photon energy. The angular resolution was 0.2°; the energy resolution was better than 25 meV over the entire range of photon energy.

Regarding a test of the robustness of the topological surface state, single crystals of Sb_2_Te_2_Se were cleaved *ex situ* in air, followed by transfer to the load-lock chamber after 5 min. After pumping for 1 h, the cleaved crystal was transferred to the analysis chamber for the ARPES experiment.

## Additional Information

**How to cite this article**: Lee, C.-K. *et al*. Robustness of a Topologically Protected Surface State in a Sb_2_Te_2_Se Single Crystal. *Sci. Rep.* **6**, 36538; doi: 10.1038/srep36538 (2016).

**Publisher’s note**: Springer Nature remains neutral with regard to jurisdictional claims in published maps and institutional affiliations.

## Supplementary Material

Supplementary Information
