# Supplementary material for: Robustness of a Topologically Protected Surface State in a Sb2Te2Se Single Crystal
Source: Sci Rep. 2016 Nov 18;6:36538. doi: 10.1038/srep36538 (PMC5114659; doi:10.1038/srep36538)
Supplement: Supplementary Information [file srep36538-s1.pdf]

Supplementary Material for

Robustness of a Topologically Protected Surface State in a Sb_2Te_2Se Single Crystal

Chao-Kuei Lee[1,2*], Cheng-Maw Cheng[2,3*], Shih-Chang Weng[3], Wei-Chuan Chen[3], Ku-Ding Tsuei[3], Shih-Hsun Yu[4], Mitch Ming-Chi Chou[4*], Ching-Wen Chang[2], Li-Wei Tu[2,5], Hung-Duen Yang[2], Chih-Wei Luo[6], and Marin M. Gospodinov[7]

[1]*Department of Photonics, National Sun Yat-sen University, 70, Lienhei Road, Kaohsiung 80424, Taiwan*
[2]*Department of Physics, National Sun Yat-sen University, 70, Lienhei Road, Kaohsiung 80424, Taiwan*
[3]*National Synchrotron Radiation Research Center, Hsinchu 30076, Taiwan*
[4]*Department of Materials and Optoelectronics Science, National Sun Yat-sen University, 70, Lienhei Road, Kaohsiung 80424, Taiwan*
[5]*Department of Medical Laboratory Science and Biotechnology, Kaohsiung Medical University, Kaohsiung 80708, Taiwan*
[6]*Department of Electrophysics, National Chiao Tung University, Hsinchu 300, Taiwan*
[7]*Institute of Solid State Physics, Bulgarian Academy of Sciences, Blvd. TzarigradskoChaussee 72, 1784 Sofia, Bulgaria*

**To whom correspondence should be addressed. E-mail: chuckcklee@yahoo.com, makalu@nsrrc.org.tw and mitch@faculty.nsysu.edu.tw*

1. Sample preparation and characterization

Single crystals of Sb_2Te_2Se were grown in a home-made resistance-heated floating-zone furnace (RHFZ). This apparatus employs a method similar to directional solidification, in which a small region of a fed rod is melted and this molten zone is moved along the crystal. The molten region moves the impurities to one end of the fed rod and leaves behind it a wake of purer material, solidified, as it moves through the ingot; the impurities concentrated in the melt have an appreciable difference of concentration between the solid and liquid phases at equilibrium. Atopological insulator (TI) crystal grown with the RHFZ method has a much more uniformcrystallinity than that from a traditional vertical Bridgman technique.

Sb_2Te_3 and Sb_2Te_2Se crystallize in the tetradymite structure with space group $R_{\underline{3}m}$. In Sb_2Te_3, the hexagonal cell comprises 15 layers grouped in 3 quintuple layers, Te^2-Sb-Te^1-Te^1-Sb,in which Te^1 and Te^2 denote the tellurium atoms at distinct Wyckoff points. In Sb_2Te_2Se, selenium atoms replace the Te^2 tellurium atoms and the lattice parameter along hexagonal axis *c*contracts. Figure S1 shows a*L*-scan along the surface normal [0 0 0 *L*] direction of Sb_2Te_3 and Sb_2Te_2Se. The present crystals exhibit high crystallinity and greatuniformity according to X-ray diffraction (XRD). Our XRD data show that the mean value of lattice parameter*c* of Sb_2Te_3 is 30.428 Å and lattice parameter*c* of Sb_2Te_2Se is 29.869 Å, consistent with the literature[1].

For further confirmation of the crystalline quality and properties, micro-Raman spectra wererecorded(Horiba Jobin-Yvon T64000 triple-grating system, CCD detector)[2]. An Ar^+ ion laser provided excitation atwavelength 514.5 nm; the beam passed through a microscope and was focused on the sample with a laser spot of micrometre size in a back-scattering geometry. A quarter-wave plate was placed in the path of the laser beam to convert the linearly polarized laser light into circularly polarized light. Stokes shifts were measured resulting from phonon emission in the inelastic scattering of the samplesnear 23 ^{o}C.

Figure S2 shows the Raman spectrum of a Sb_2Te_2Se single crystal; the active Raman modes are observed with wavenumbers in the range 20–225 cm^{-1}. A_{1g}^1 (82.2 cm^{-1}) and A_{1g}^2 (188.9 cm^{-1}) modes are longitudinal vibrations along axis*c* of the crystal, at which, with neighboring atoms of groups V and VI moving in the same direction, the A^1 mode has a smaller wavenumber than the A^2 mode in which the atoms move in the opposite direction. E_g^1 (41.1 cm^{-1}) and E_g^2 (117.5 cm^{-1}) modes are lateral vibrations in the plane vertical to axis*c*, with the E^2 mode having a largerwavenumber shift than the E^1mode because thegroup V and VI atoms move in opposite directions whereas in the same direction for the E^1 mode. An extra Raman

shift, E^H(Te) resulting from Te bonding, is observed at 138.3 cm^{-1}, which disappeared gradually whenSewas added to the Sb-Se-Te material system replacing Te[3]. Concurrently, the A^1(Te) mode is located about the side of the E_g^2 mode and the E^L(Te) mode emerges on the side of the A_{1g}^1 mode of greater energy.

2. Sb_2Te_2Sedoped with alkali metal

Photoemission spectra were collected in a UHV chamber equipped with a hemispherical analyzer (Scienta R4000) with collecting angle ±15°. The polarization vector was invariably in the angular dispersive plane. The single crystals were cleaved *in situ* and measured at sample temperature 83 K, with photon energy 24 eV and energy resolution 14 meV. The base pressure in all used chambers was less than 6.5×10^{-11}Torr. Potassium (K) and caesium atoms were deposited from a well calibrated SAES getter source at 83 K. The amount of deposited K atoms was estimated from the occupied area of the 2D Fermi surface,on mapping the area of the surface BZ in a bilayer graphene (BLG)/SiC substrate, and the core level photoemission spectra of potassium 3*p*. For K deposited for 5 min, the amount of charge transfer on BLG/SiC is about 0.012 electrons/per unit cell. Figure S3a displays a comparison of photoemission plots ofundoped and doped Sb_2Te_2Se crystals. Figure S3b shows the photoemission spectrumat normal emission with varied duration of deposition. That the peak position of theresonance surface state (RSS) moved toward increased binding energy about 83 meV after deposition for 5 min implies a*n*-type doping behavior. With increasing deposition of K, no alteration of the peak position of the RSS was observed, but the energy distribution curve (EDC) became broadenedbecause of surface pollution. Figure 3c shows MDC plots at the Fermi level for undoped and doped Sb_2Te_2Se crystals. The Fermi vector k_F ~ 0.048 $Å^{-1}$in doped

Sb_2Te_2Se is smaller than that,0.065 Å^{-1}, in an undoped crystal.We observed no Dirac point because of the effect of saturated potassium doping, but the position of the Dirac point was determined at 237meV above the Fermi level through an extrapolation of the linear dispersion of the topological surface state (TSS). The data from theexperiment with caesium doping is consistent with the result of the Sb_2Te_2Se crystaldopedwith potassium.

3. Experiment on Sb_2Te_2Sedependenton photon energy

In the photoemission experiment, the normal component of electron wave vector $k_\perp$ of the initial state is based on a free-electron model in the final state[4],

$$k_\perp = \sqrt{\frac{2m}{\hbar^2}\left(E_{kin}\cos^2\theta + V_0\right)} \qquad (1)$$

in which E_{kin} is the kinetic energy of the photoelectron and V_0 is the inner potential. To probe the property of a 3D bulk-valence band, we performed an ARPES experiment dependent on photon energy in a range from 12 to 50 eV. In the Bi_2Se_3 compound, the value 9.7 eV of the inner potential of Bi_2Se_3 is determined from the top of the valence band and the bottom of the conduction band observed at photon energy 19 eV. We carefully examined our data in normal emission with varied photon energy. The top of the valence band at normal emission in Sb_2Te_2Se appears at either 18 eV or 19 eV. A reasonable value of the inner potential of Sb_2Te_2Se is between 10.64 and 9.64 eV. A *k*-space map in the bulk Brillouin zone (BZ) plotted with varied photon energyand collecting angle $\pm 18^\circ$ of the detector was used to explore the k_z dependence of TSS, RSS and bulk bands, as shown in FS5b. Arange of photon energy from 12 eV to 42 eV is expected to cover several periods of the Brillouin zone in thek_z direction. Figure S4 displays the band-mapping plots of the ARPES spectra in

Sb_2Te_2Se (0001) along direction $\bar{\Gamma}-\bar{K}$ with photon energies varied from 12 eV to 24 eV. Figure S5a displays the band-mapping plots of ARPES spectra in Sb_2Te_2Se (0001) along direction $\bar{\Gamma}-\bar{M}$ with photon energies varied from 18 eV to 42 eV. Figure S6 displays the MDC plots at the Fermi level along direction $\bar{\Gamma}-\bar{M}$ with varied photon energy. No contribution was observable from the bulk valence band in the middle of the $\bar{\Gamma}-\bar{M}$ branch.Figure S7shows a series of EDC in the Sb_2Te_2Se crystal in direction $\bar{\Gamma}-\bar{M}$ with varied photon energy.As shown in figure S7, the edge of the valence band in the middle of$\bar{\Gamma}-\bar{M}$labeled with a black dashed line is clearly below the Fermi level. This observed behavior shows no contribution at the Femi level in the middle of $\bar{\Gamma}-\bar{M}$, which is consistent with the analysis of the MDC.

According the *k*-space map the bulk BZ and period pf k_z, the band mapping result at 18 eV and 22 eV can correspond to the result at 30 or 32 eV and 36 eV.To examine the bulk band crossing at theΓpoint, EDC and MDC plots near the Fermi level at the Γ point with 18, 22, 30 and 32 eV are shown in figs.S4b and S4c.No obvious bulk band dispersion is observed in the plots.In figure S5c, the second derivative plot of photoemission intensity mapping at 22 and 36 eV have similar band structure due the periodic property of k_z, but no obvious bulk and dispersion was also observed in the MDC plots at 22 eV and 36 eV.

4. Fourier-transform infrared spectra(FTIR)

A commercial Fourier-transform infrared (FTIR) spectrometer (Cary 660 FTIR Spectrometer, Agilent Technology) was employed to perform temperature-dependent mid-infrared absorption measurements. Free-standing Sb_2Te_2Se samples were cleaved to be thin enough for measurements, but the actual thicknesses of the used samples are unknown. As shown in Fig. S8a, the absorption edge (2750 cm^{-1}, 341meV) at room temperature (296 K) is higher than the theoretical prediction[6]. As the

temperature decreases, the absorption edge becomes clearer and trends to 417meV (3359 cm^{-1}) at 98 K.

5. Measurement of resistivity transport

To measure the transport properties of Sb_2Te_2Se and Sb_2Te_3 compounds, we used silver paint and gold wires to connect to the cleaved crystal. Acommercial physical property measurement system (Quantum Design PPMS Model 6000) was used to measure the temperature dependence of resistivity with a conventional four-wire system. As shown in figure S9, the resistivity of Sb_2Te_2Se is clearly about ten times that of Sb_2Te_3 and the extracted carrier concentration ~ $3.1x10^{19}$ cm^{-3} of Sb_2Te_2Se is less than that ~ $2.2x10^{20}$ cm^{-3} of Sb_2Te_3.

References

1. Anderson, T. L. & Krause, H. B. Refinement of the Sb_2Te_3 and Sb_2Te_2Se structures and their relation to nonstoichiometric $Sb_2Te_{3-y}Se_y$ compounds. ActaCryst. B**30**, 1307-1310 (1974).
2. Hsiao, C.-L. *et al*. Micro-Raman spectroscopy of a single free-standing GaNnanorod grown by molecular beam epitaxy. Appl. Phys. Lett. **90**, 043102 (2007).
3. Misochko, O. V. *et al.* Polarization dependence of coherent phonon generation and detection in the three-dimensional topological insulator Bi_2Te_3. Phys. Rev. B **91**, 174303 (2015).
4. Damascelli, A. Probing the electronic structure of complex systems by ARPES. PhysicaScripta**T109**, 61-74 (2004).
5. Tanaka, Y. *et al.*Evolution of electronic structure upon Cu doping in the topological insulator Bi_2Se_3, Phys. Rev. B **85**, 125111 (2012).
6. Lin, H. *et al.* An isolated Dirac cone on the surface of ternary tetradymite-like topological insulators. New Journal of Physics **13**, 095005 (2011).

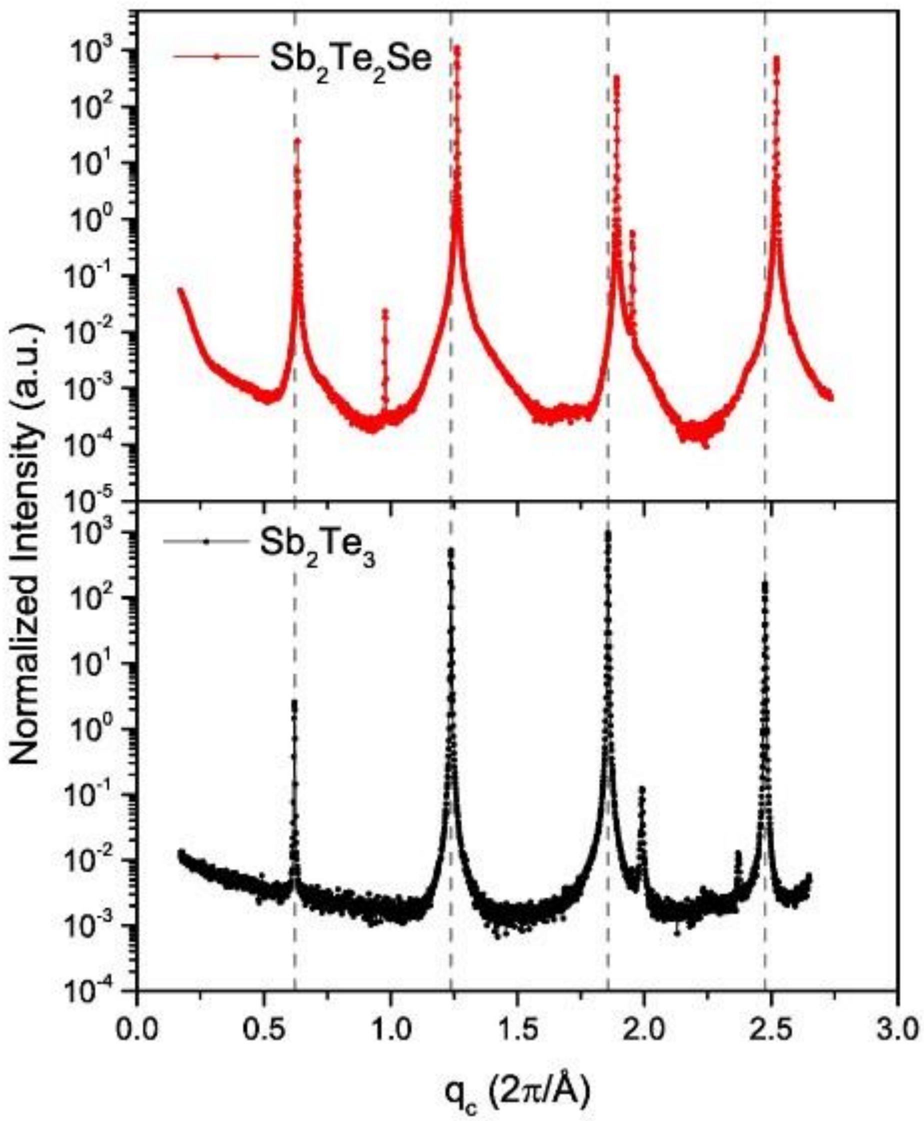

Figure S1 |*L*-scan in high-resolution X-ray diffraction measurements of Sb_2Te_3 and Sb_2Te_2Se.Bulk crystals Sb_2Te_3 and Sb_2Te_2Se with orientation [0001] were used for this experiment. The $\boldsymbol{\theta}$-2$\boldsymbol{\theta}$ scans along *c** of Sb_2Te_3 and Sb_2Te_2Se are shown in the figure as indicated. The four major signals indicate reflections (0 0 0 3), (0 0 0 6), (0 0 0 9) and (0 0 0 12); the dashed lines show the positions of signalsof Sb_2Te_3.

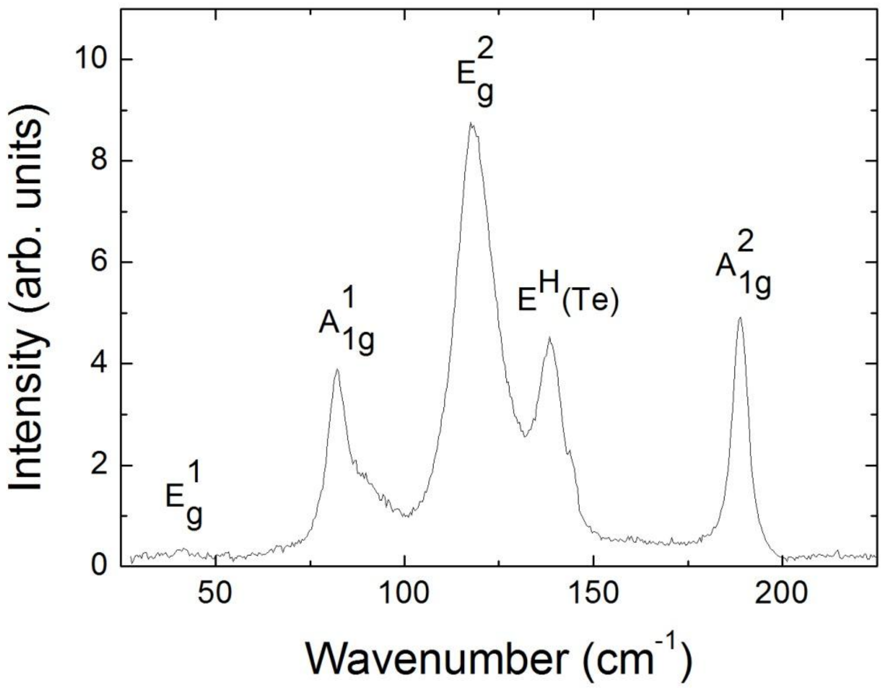

Figure S2 | Raman Stokes modes of Sb_2Te_2Se near 296 K.

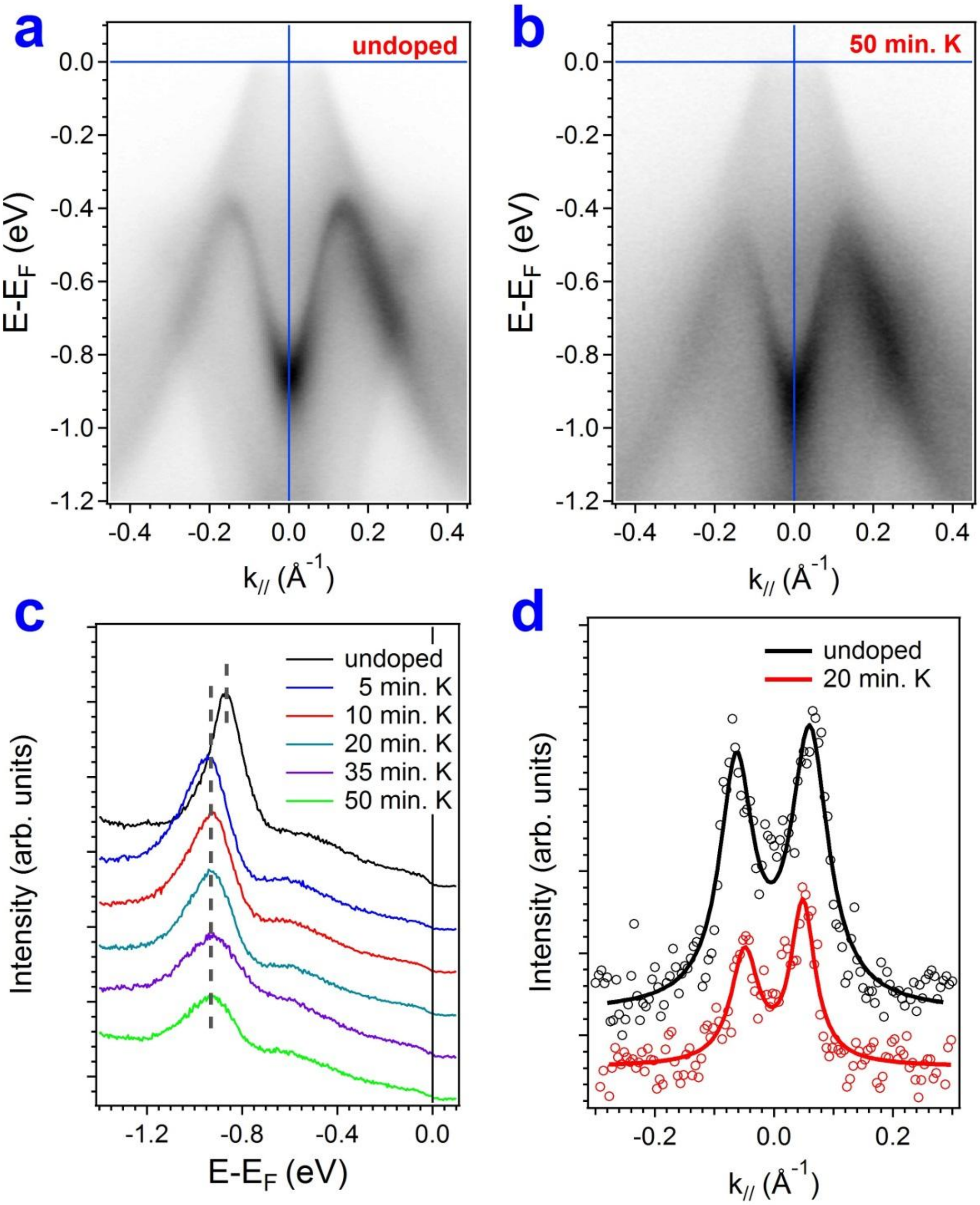

Figure S3 | Electronic structure of potassium-doped Sb_2Te_2Se.a, Image of ARPES spectra of undoped Sb_2Te_2Se in direction $\bar{\Gamma}-\bar{K}$. **b**, Image of ARPES spectra of potassium-doped Sb_2Te_2Se in direction $\bar{\Gamma}-\bar{K}$. **c**, Comparison of EDC in the normal emission for undoped and doped Sb_2Te_2Se crystals. The position of the maximum of the RSS moves to increased binding energy after deposition for 5 min, but no further change was observed withprolonged deposition. **d**, MDC at the Fermi level for undoped and doped Sb_2Te_2Se crystals. The smaller value of k_F in doped Sb_2Te_2Se crystal indicates a*n*-type doping behavior.

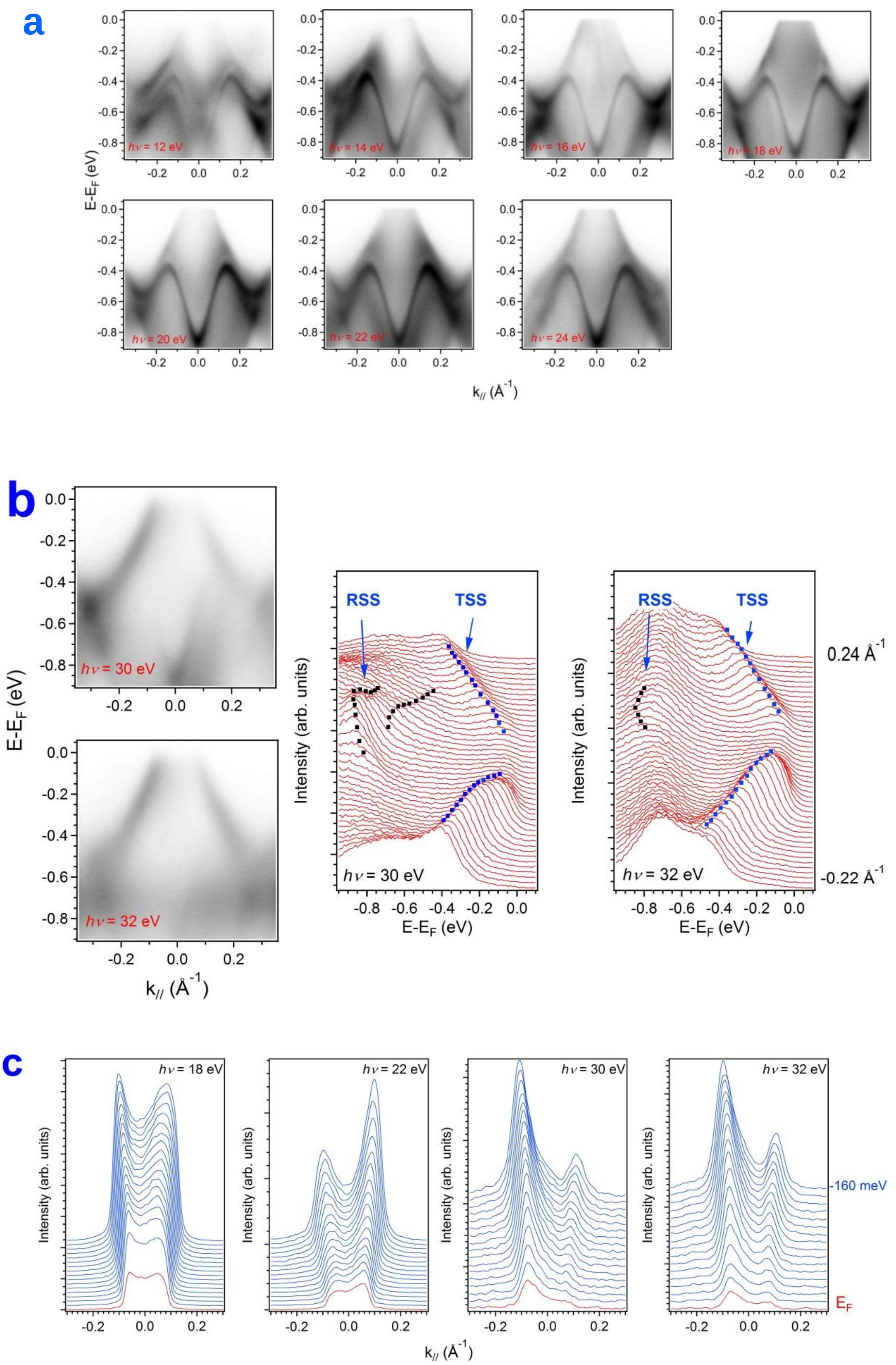

Figure S4 | Results of band mapping in a Sb_2Te_2Se crystal in direction $\bar{\Gamma} - \bar{K}$ with varied photon energy.a, Band mapping images with varied photon energy. **b,**

Band mapping images and EDC plots with 30 and 32 eV.**c,**MDC plots around the Fermi level at the Γpoint with 18, 22, 30 and 32 eV. No obvious bulk band dispersion is observed around the Γ point near the Fermi level in the EDC and MDC plots. A flat shaded area existed between -0.6 eV and -0.8 eV at 32 eV plot in **c** can be attributedas Sb 4*d* due to high order light.

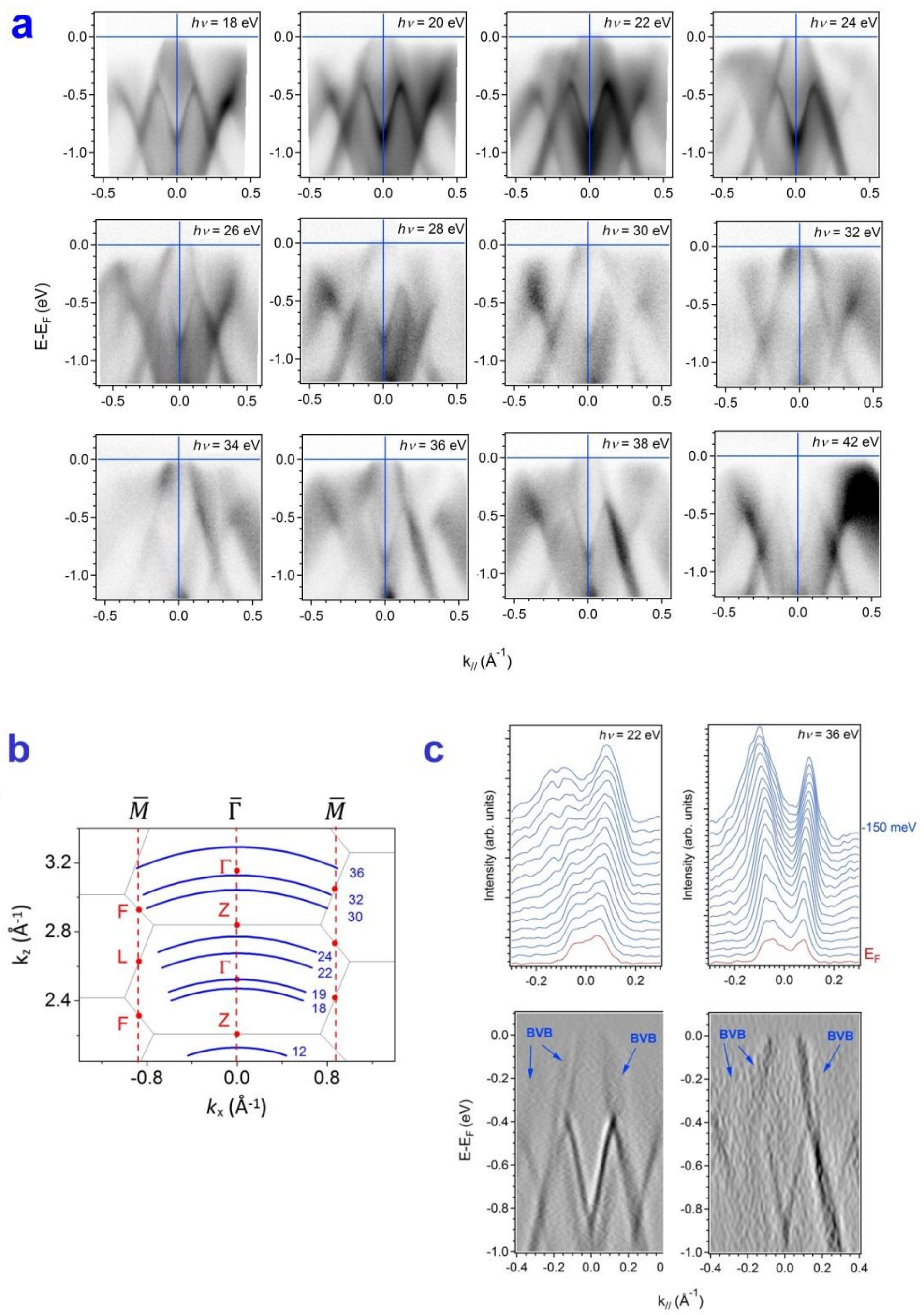

Figure S5 | Results of band mapping in a Sb_2Te_2Se crystal in direction $\bar{\Gamma}-\bar{M}$ with varied photon energy.a, Band mapping images with varied photon energies.**b,**a k-space map in the bulk Brillouin zone (BZ) plotted with varied photon energyand collecting angle ± 18 of the detector. **c,** Upper: MDC plots around the Fermi level at the Γ point with 18, 22, 30 and 32 eV. Bottom: Second derivative plot of photoemission intensity mapping at 22 and 36 eV.

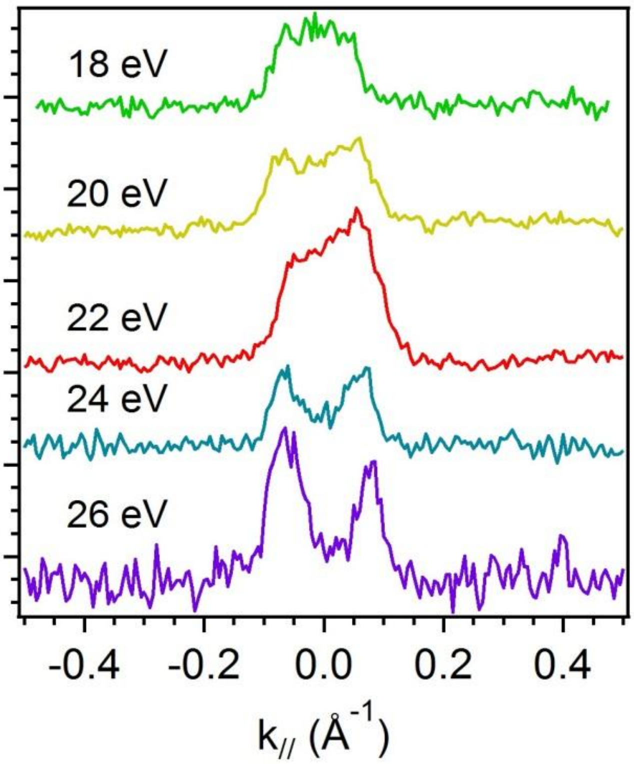

Figure S6 | MDC at the Fermi level in Sb_2Te_2Se crystal in direction $\bar{\Gamma} - \bar{M}$ with varied photon energy. No contribution was observable from the bulk valence band in the middle of the $\bar{\Gamma}\bar{M}$ branch.

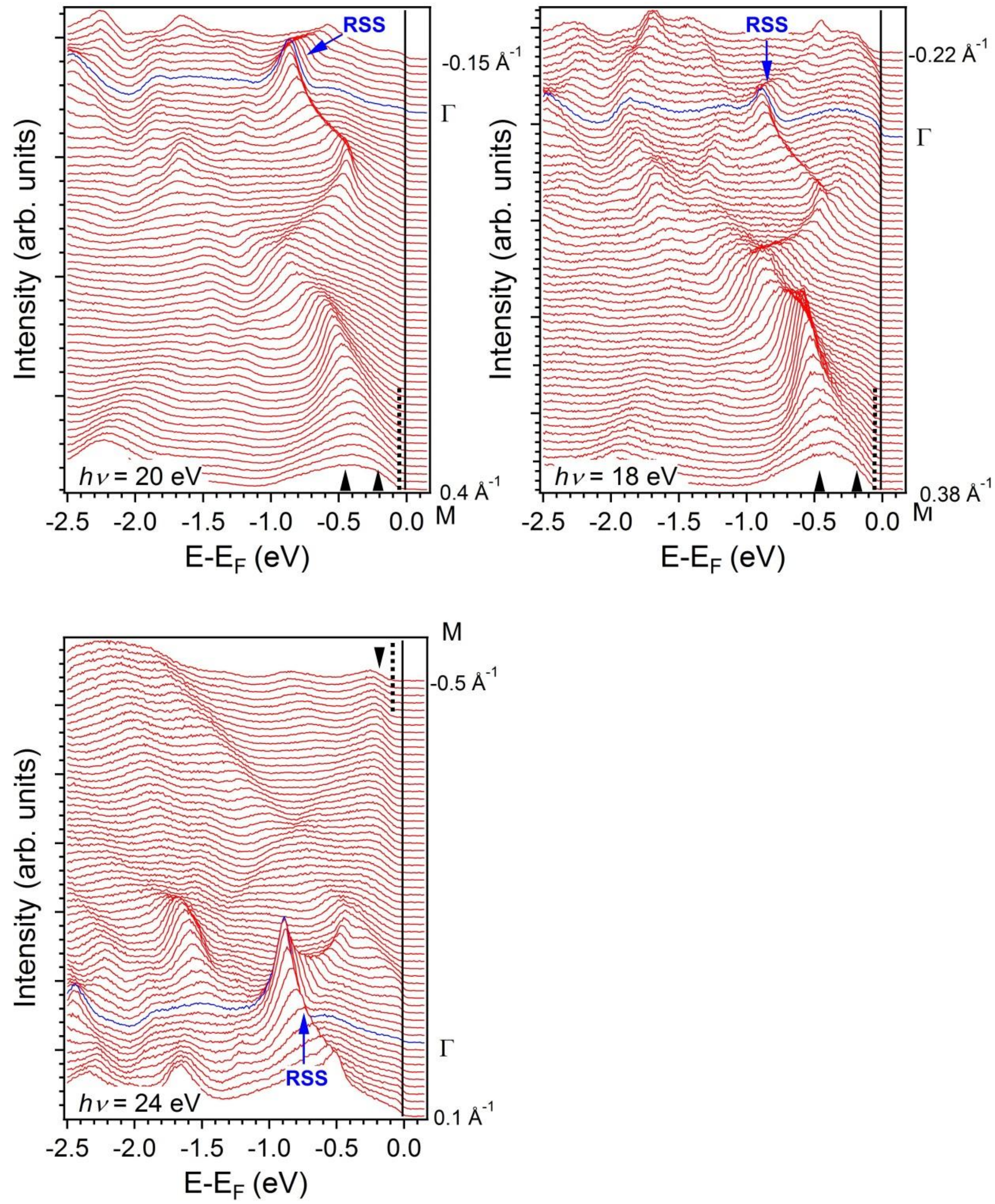

Figure S7 | EDC in Sb_2Te_2Se crystal in direction $\bar{\Gamma} - \bar{M}$ with varied photon energy. As shown the dash line in each plot, no contribution was observable from the bulk valence band in the middle of the $\bar{\Gamma}\bar{M}$ branch.

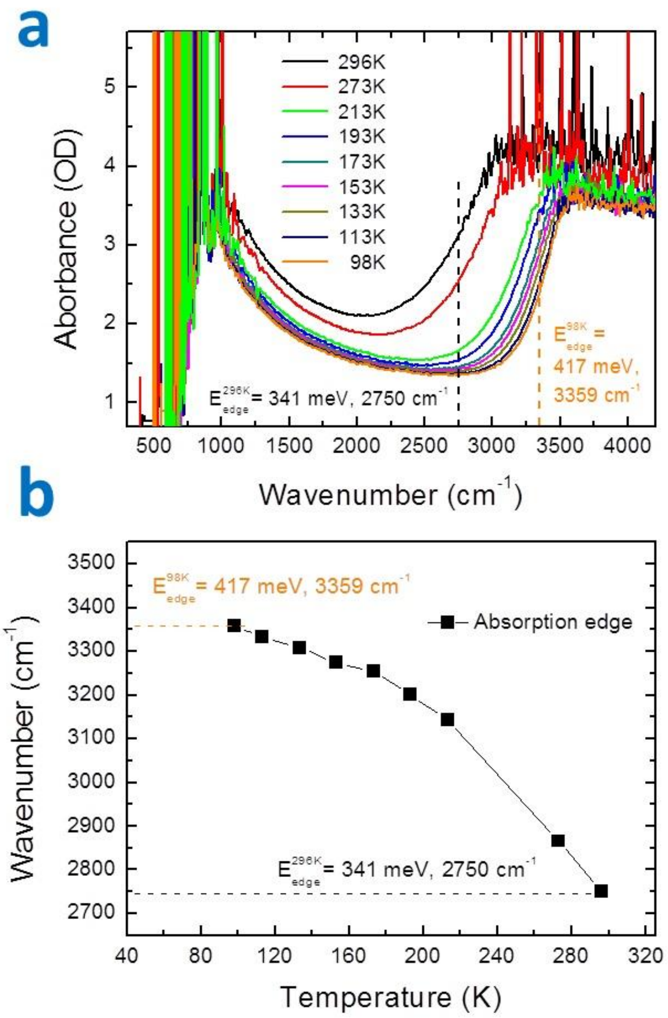

Figure S8 | FTIR spectra of free-standing Sb_2Te_2Se samples at low temperature.a, Mid-infrared absorption spectra of free-standing Sb_2Te_2Se were measured in a range 500-4200 cm^{-1}. The middle of the edges is chosen as the absorption edge. **b**, The temperaturedependence of absorption edge.

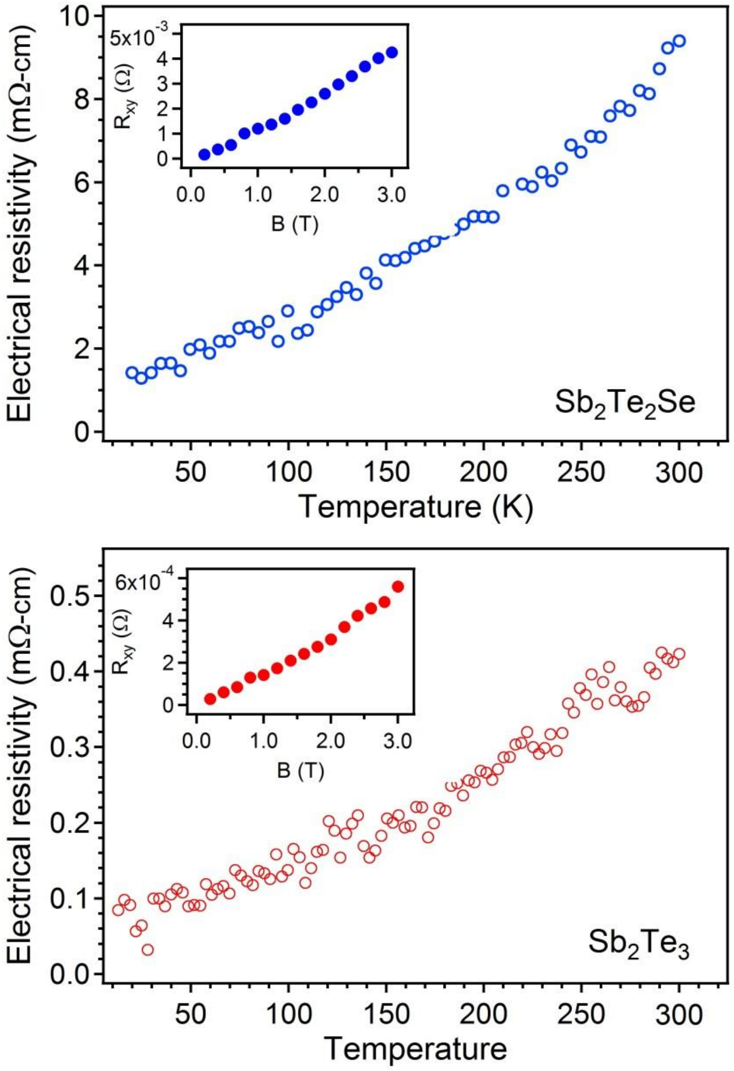

Figure S9 | The temperature dependence of resistivity and Hall measurement at RT with Sb_2Te_2Se and Sb_2Te_3 single crystals. The extracted carrier number is 3.1×10^{19} cm^{-3} and 2.2×10^{-20} cm^{-3} for Sb_2Te_2Se and Sb_2Te_3 respectively.
